# Dietary Effect on the Proteome of the Common Octopus (*Octopus vulgaris*) Paralarvae

**DOI:** 10.3389/fphys.2017.00309

**Published:** 2017-05-17

**Authors:** Inmaculada Varó, Gabriel Cardenete, Francisco Hontoria, Óscar Monroig, José Iglesias, Juan J. Otero, Eduardo Almansa, Juan C. Navarro

**Affiliations:** ^1^Instituto de Acuicultura Torre de la Sal (CSIC), Ribera de CabanesCastellón, Spain; ^2^Departamento de Zoología, Universidad de GranadaGranada, Spain; ^3^Faculty of Natural Sciences, Institute of Aquaculture, University of StirlingStirling, Scotland; ^4^Centro Oceanográfico de Vigo, Instituto Español de OceanografíaVigo, Spain; ^5^Centro Oceanográfico de Canarias, Instituto Español de OceanografíaSanta Cruz de Tenerife, Spain

**Keywords:** *Octopus vulgaris*, paralarvae, nutritional stress, proteome, novel biomarkers, welfare

## Abstract

Nowadays, the common octopus (*Octopus vulgaris*) culture is hampered by massive mortalities occurring during early life-cycle stages (paralarvae). Despite the causes of the high paralarvae mortality are not yet well-defined and understood, the nutritional stress caused by inadequate diets is pointed out as one of the main factors. In this study, the effects of diet on paralarvae is analyzed through a proteomic approach, to search for novel biomarkers of nutritional stress. A total of 43 proteins showing differential expression in the different conditions studied have been identified. The analysis highlights proteins related with the carbohydrate metabolism: glyceraldehyde-3-phosphate-dedydrogenase (GAPDH), triosephosphate isomerase; other ways of energetic metabolism: NADP^+^-specific isocitrate dehydrogenase, arginine kinase; detoxification: glutathione-S-transferase (GST); stress: heat shock proteins (HSP70); structural constituent of eye lens: S-crystallin 3; and cytoskeleton: actin, actin-beta/gamma1, beta actin. These results allow defining characteristic proteomes of paralarvae depending on the diet; as well as the use of several of these proteins as novel biomarkers to evaluate their welfare linked to nutritional stress. Notably, the changes of proteins like S-crystallin 3, arginine kinase and NAD^+^ specific isocitrate dehydrogenase, may be related to fed vs. starving paralarvae, particularly in the first 4 days of development.

## Introduction

The common octopus (*Octopus vulgaris*) is one of the most important cephalopod species recommended for European aquaculture diversification due mainly to its rapid growth, elevated food conversion index, and high market demand and price (Navarro and Villanueva, 2000; Vaz-Pires et al., 2004; Iglesias et al., 2007). Nowadays its culture is hampered by massive mortalities (Iglesias and Fuentes, 2014) occurring during early life-cycle stages, (paralarvae), representing the main obstacle for commercial production of this species (Iglesias et al., 2007, 2014; Villanueva and Norman, 2008). In fact, the life cycle of *O. vulgaris* under captivity conditions was completed for the first time in 2001 (Iglesias et al., 2004), but until now, it has not been possible to successfully rear the paralarvae up to juveniles and sub-adults with acceptable survivals for the commercial production of this species (Rodríguez and Carrasco, 1999; Móxica et al., 2002; Iglesias et al., 2014). The actual causes of such mortality remain unknown, although failure to fulfill the dietary requirements for key nutrients particularly essential lipids appear to be a major factor (Navarro et al., 2014). Thus, substantial efforts have been made to associate dietary lipids to mortality and growth at paralarval stages (Navarro and Villanueva, 2000, 2003; Seixas et al., 2010; Monroig et al., 2012a,b; Navarro et al., 2014), with meta-analysis techniques pointing out clearly at this link (Garrido et al., 2016a). However, the lack of substantial differences in performance between the experimental dietary regimes and control treatments in paralarval cultures fed diets with different essential lipid profiles suggested that other not yet explored factors account for the high mortality mentioned above. Thus, the zootechnical productions may undergo some unspecific stress that adds up to the putative nutritional deficiencies contributing very significantly to reduced paralarval welfare manifested in very low survival and depressed growth.

Like for most early stages of marine fish and crustaceans, feeding is based in the use of live preys. *Artemia* nauplii and metanauplii are extensively used as food for availability reasons, but crustacean zoeae (*Maja, Pagurus, Grapsus*) seem a more suitable and natural prey from the view point of their nutritional composition, and have been used with some success (Iglesias et al., 2004, 2014; Navarro et al., 2014; Garrido et al., 2016a). Recently, the culture in big volumes (1,000 L tanks) with the use of ongrown *Artemia* biomass using microalgae, like *Nannochloropsis* or *Isochriysis*, as food (Iglesias et al., 2004, 2006; Fuentes et al., 2011) have moderately improved paralarval survival, but still far from industrial scenarios. To the nutritional stress caused by inadequate and/or unbalanced diets (Iglesias et al., 2007; Garrido et al., 2016b), and lack of optimal reproducible culture protocols, it should be added that there is little knowledge on paralarvae physiology and behavior allowing to understand the poor culture performance. In fact, studies on the basic processes involved in nutritional and physiological stress are limited in cephalopod paralarvae. Under such an unmanageable panorama, omics technologies, specifically proteomic approaches come at hand.

Proteins form a major class of macronutrients, because they participate in every cellular process. Therefore, the global profiling of the proteome, defined as the entire protein complement of the genome expressed at a particular time, offers the potential for identification of important biomarkers of nutritional state that respond to alterations in diet. Currently, nutritional research is taking advantage of proteomics technologies to discover biologically active food components, to assess their quality and safety, and to demonstrate their biological efficacy. For example, using proteomics, it was shown that in mice, the consumption of different dietary oils induced either differential expression of long chain acyl-CoA thioester hydrolase protein as an indicator of β-oxidation of fatty acids in the liver, or differential expression of adipophilin protein as an indicator of selective hepatic lipid accumulation and triglyceride secretion (Roos and McArdle, 2008). Proteomics was also applied to explain the mechanisms underlying changes in hepatic lipid metabolism of rats during zinc deficiency (Tom Dieck et al., 2005 cited by Roos and McArdle, 2008). Regarding fish species, zebrafish proteome was altered by calorie restriction (Jury et al., 2008) and rainbow trout proteome was found to reflect dietary manipulations (Martin et al., 2003). Thus, application of proteomics techniques to “map” and read metabolic dysfunctionalities of octopus paralarvae seem a more than promising field.

Up to date, all previous studies of nutritionally-derived stress in paralarvae cultures of the common octopus have been carried out using conventional approaches, testing the effects of a variable on potential (bio)markers of such effects (Garrido et al., 2017). Global protein profiling offers the potential of comparing control vs. treated animals (or the effects of different dietary treatments) beyond the frame of an aprioristic approach, in the search of highlighted differences that can help to establish clues about the metabolic pathways affected. We report here on an experiment analyzing the proteomes of *O. vulgaris* paralarvae, comparing the effect of fasting during the early days of development, as well as the response of two dietary treatments based on either enriched *Artemia* metanauplii or crustacean zoeae as live preys.

## Materials and methods

### Paralarval rearing

*O. vulgaris* paralarvae were obtained from a broodstock kept at the Spanish Institute of Oceanography IEO (Vigo), following the rearing conditions described by Móxica et al. (2002). Paralarvae were raised up in black cylindrical 500 L tanks until 16 days, before massive mortalities start. The initial paralarvae density was 10 individuals L^−1^ (5,000 individuals per tank). A closed water circuit was used during the first 5 days and partly opened (4 h/day) until the end of the experiment. The temperature was kept at 21–23°C, and the salinity at 35 psu. Central aeration and drainage were used for water renovations and surface cleaners based on air pressure were applied. The light intensity in the tank surface was of 800–1,000 lux during 24 h.

Two dietary treatments were tested. *Artemia* group (A) consisted of paralarvae fed *Artemia* nauplii (Sep-Art EG, INVE Aquaculture, Belgium) enriched with the microalgae *Nannochloropsis* sp. and *Isochrysis galbana* at 0.5 individuals mL^−1^ per day. Zoeae group (Z) consisted of paralarvae fed live crustacean zoeae (*Maja brachydactyla*) at 0.01 Maja zoeae mL^−1^ per day in co-feeding with (A). Co-feeding was unavoidable from the evidence that the production of Maja zoeae did not suffice to keep a prey density equivalent to treatment A. Thus, every effort was made to try to keep similar prey densities in both treatments. Also, an unfed paralarvae group, named (I), was kept from hatchling to day 4. *M. brachydactyla* zoeae were produced as described in Iglesias et al. (2014).

Paralarvae dry weight was determined individually after oven drying for 20 h at 110°C as described in Iglesias et al. (2014). Before, animals were sacrificed in chilled seawater (−2°C) and rinsed in distilled water.

Pooled paralarval (5–10) samples were collected from each experimental group at days 0, 4, and 16 for proteomic analysis. The samples were rinsed, frozen in liquid nitrogen and stored at −80°C until analyzed. The study was exempt from ethics approval, since the zootechnical experiments were performed in 2013 before the Spanish Legislation made it compulsory by established Ethical Committees in the Research Institutions. The experiments were conducted under ethical protocols and recommendations that are nowadays fully compliant with the European directive (2010/63/EU), the Spanish law (RD 53/2013), and the Guidelines for the Care and Welfare of Cephalopods in Research (Fiorito et al., 2015).

### 2D differential in gel electrophoresis (2D-DIGE): sample preparation and protein labeling

Proteins from samples were directly extracted in DIGE lysis buffer (7 M urea, 2 M thiourea, 4% CHAPS, 30 mM Tris and 1x complete protease inhibitor EDTA free, Roche) using the 2D grinding kit system (General Electric Healthcare). The solubilized proteins were separated from non-solubilized cellular components by centrifugation (20,000 g × 20 min). Salts and any interfering components were removed using the 2D Clean-up kit (GE Healthcare) and after precipitation, proteins were resolublized in DIGE label buffer (7 M urea, 2 M thiourea, 4% CHAPS, 20 mM Tris-pH 8.5). Protein concentration was determined using the Bradford Bio-Rad Protein Assay (RcDc Kit) with bovine serum albumin (BSA) as standard.

Proteins from each experimental group were randomly labeled either with Cy3 or Cy5 following to the manufacturer's instructions (GE Healthcare). Briefly, 50 μg protein of each sample was labeled with 400 pmol CyDye DIGE Fluor minimal Dye by vortexing and incubated on ice in the dark for 60 min. The labeling reaction was stopped with 1 μL of 10 mM lysine followed by incubation on ice for 10 min. An internal standard sample was prepared by pooling 25 μg of protein from each sample, and by labeling by Cy2 as described above. Differentially labeled samples (150 μg total protein) were mixed and 65 mM DTT and 1% ampholytes (pH = 3–10 NL) were added to the mixture before running the first dimension.

### Gel electrophoresis (2D-dige gel): separation and image capture

A total of 24 protein samples (6 experimental groups × 4 biological replicates, Supplementary Table 1) were combined in pairs, and analyzed on a total of 12 2D gels following the experimental design given in Supplementary Table 2. 24 cm- IPG strips (pH = 3–11 NL) were rehydrated in 8 M urea, 4% CHAPS, DeStreak (12 μL mL^−1^), and 1% ampholytes (pH = 3–10 NL) overnight at room temperature. Cy-labeled samples were applied onto IPG rehydrated strips via anodic cup loading, and IEF (first dimension) was performed on a Ettan IPGphor II horizontal electrophoresis system (GE Healthcare) at 20°C using the following IEF protocol: step 1:300 V 4 h, gradient to 1,000 V 6 h, gradient to 8,000 V 3 h; step 2: 8,000 V until reached 32,000 V h.

After IEF, the strips were reduced in equilibration buffer [Tris 50 mM, urea 6 M, and glycerol 30% (v/v), 2% SDS (w/v)] containing 2% DTT (w/v), for 15 min at room temperature; followed by alkylation in equilibration buffer containing 2.5% (w/v) iodoacetamide, for 15 min at room temperature. The proteins were then separated (second dimension) on 12.5% acrylamide SDS-PAGE gels (25 cm × 21 cm × 1 mm) using an Ettan DaltSix Unit (GE Healthcare) electrophoresis system at 2 W per gel for 1 h and 15 W per gel for 6 h.

After electrophoresis, the 2-D gels were scanned with a Typhoon^TM^ 9400 Variable Mode Imager, at 100 μm resolution to visualize the labeled proteins. Excitation/emission wavelengths were chosen specifically for Cy2, Cy3, and Cy5 according to manufacturer's recommendations (GE Healthcare).

### Data analysis

Fluorescence images were analyzed using DeCyder™ 2D software (v.7.0) and the multivariate statistical module EDA (Extended Data Analyses; GE, Healthcare), as described in Varó et al. (2010). First, the intra-gel images were individually processed by DeCyder-DIA (Differential In-gel Analyses) software module to co-detect and differentially quantify the protein spots in the images, taking the internal standard as reference to normalize the data, and with the threshold set to 2 standard deviations. Thereafter, the DeCyder-BVA (Biological Variation Analysis) was applied to inter-gel matching, and differences in average ratios of protein expression were analyzed by the Student's *t*- test and One-Way ANOVA, with *p* ≤ 0.05 being considered significant. Finally, EDA software was used for multivariate statistical analysis of data. Principal components analyses (PCA) were carried out using an algorithm included in the EDA software, incorporating only data from proteins present in at least 90% of the spot maps and applying a *t*-test filter (*p* ≤ 0.05).

### Mass spectrometry

Protein spots showing significantly altered expression levels between groups were manually excised from analytical silver stained gels and digested with sequencing grade trypsin (Promega) as described elsewhere (Shevchenko et al., 1996). The digestion was stopped with 0.1% TFA (trifluoroacetic acid, Sigma) and the digested peptides were concentrated to 7 μL. BSA plug was analyzed in the same way to control the digestion process.

Digested samples were subjected to PMF-MS/MS (MALDI-TOF-TOF) and/or LC-MS/MS analyses.

#### PMF-MS/MS (MALDI-TOF-TOF) analysis

Previously, MALDI plate and the acquisition methods were calibrated with 0.5 μL of the CM5 calibration mixture (ABSciex) in 13 positions. The resulting mixtures were analyzed in a 5800 MALDI- TOF-TOF (ABSciex) in positive reflection mode (3,000 shots in every position). Five of the most intense precursors (according to the threshold criteria: minimum signal-to-noise: 10, minimum cluster area: 500, maximum precursor gap: 200 ppm, maximum fraction gap: 4) were selected for every position for the MS/MS analysis, and data was acquired using the default 1 kV MS/MS method. The MS and MS/MS information was sent to MASCOT via the Protein Pilot (v 4.5 ABSciex) to be identified.

#### LC-MS/MS analysis

Protein spots without a positive identification were analyzed by LC-MS/MS. 5 μL of each sample were loaded onto a trap column (NanoLC Colum, 3 μ C18-CL, 350 μm × 0.5 mm; Eksigen) and desalted with 0.1% TFA at 3 μL/min for 5 min. Then, peptides were loaded onto an analytical column (LC Column, 3 μm C18-CL, 75 μm × 12 cm, Nikkyo) and equilibrated in 5% acetonitrile (ACN), 0.1% formic acid (FA). The peptide elution was carried out with a linear gradient of 5–45% B in A for 15 min (A: 0.1% FA; B: ACN, 0.1% FA) at a flow rate of 300 nL/min. Peptides were analyzed in a nanoESI qQTOF mass spectrometer 5600 Triple TOF (ABSciex). The tripleTOF was operated in information-dependent acquisition mode, followed by 0.05-s product ion scan from 100 to 1,500 m/z on the 50 most intensive 2–5 charged ions. LC-MS/MS information was analyzed using Protein Pilot search engine software (v.4.5; ABSciex).

### Protein identification

The PMF search was performed on NCBI databases. Searches were done with tryptic specificity allowing one missed cleavage and a tolerance on the mass measurement of 100 ppm in MS mode and 0.8 Da for MS/MS mode. Carbamidomethylation of Cys was used as a fixed modification and oxidation of Met and deamidation of Asn and Gln as variable modifications.

For LC-MS/MS data Protein Pilot search engine software (v.4.5 ABSciex) was used. Protein Pilot default parameters were used to generate a peak list directly from 5600 TripleTof wiff files. The Paragon algorithm of Protein Pilot was used to search NCBI protein database with the following parameters: trypsin specificity, iodoacetamidecys-alkylation, no taxonomy restriction, and the search effort set to rapid.

For ESTs identifications, the MGF (mascot generic file) generated by Protein Pilot were sent to MASCOT via the Deamon software (Matrix Science). Database search was performed on ESTs database: EST_cephalopoda cephalopoda_140224 (684204 sequences; 143385132 residues). Searches were done with tryptic specificity allowing one missed cleavage and a tolerance on the mass measurement of 50 ppm in MS mode and 0.6 Da in MSMS mode. Carbamidomethylation of Cys was used as a fixed modification and oxidation of Met and deamidation of Asn and Gln as variable modifications.

## Results

### Dry weight

The dry weight of paralarvae at hatching was 0.333 ± 0.080 mg. At 4 days unfed paralarvae decreased their weight to 0.177 ± 0.070 mg, whereas values of 0.411 ± 0.014 mg and 0.367 ± 0.033 mg were reached by Z and A groups. At 16 days, these raised again to 0.713 ± 0.062 mg and 0.621 ± 0.051 mg respectively.

### DIGE analyses and protein identification

**Figure 1:**
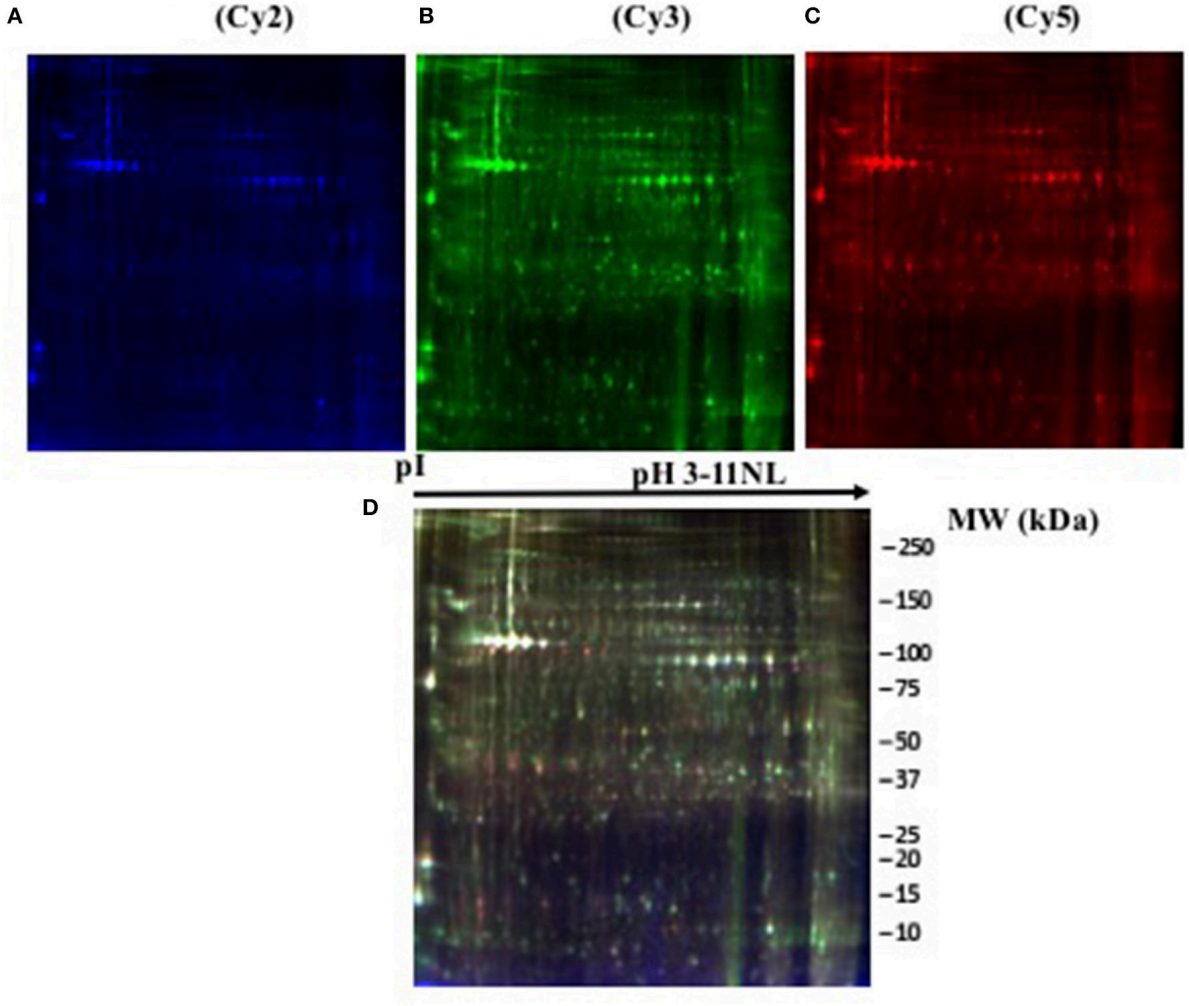
**Representative two-dimensional differential in gel electrophoresis (2D-DIGE) of *Octopus vulgaris* paralarvae proteins corresponding to condition 1**. Gel was scanned to obtain single images corresponding to: **(A)** (Cy2, blue), **(B)** (Cy3, green), **(C)** (Cy5, red) labelled samples, and **(D)** (overlay gel image of the three dyes). Details in section 2D Differential in Gel Electrophoresis (2D-DIGE): Sample Preparation and Protein Labeling.

**Figure 2:**
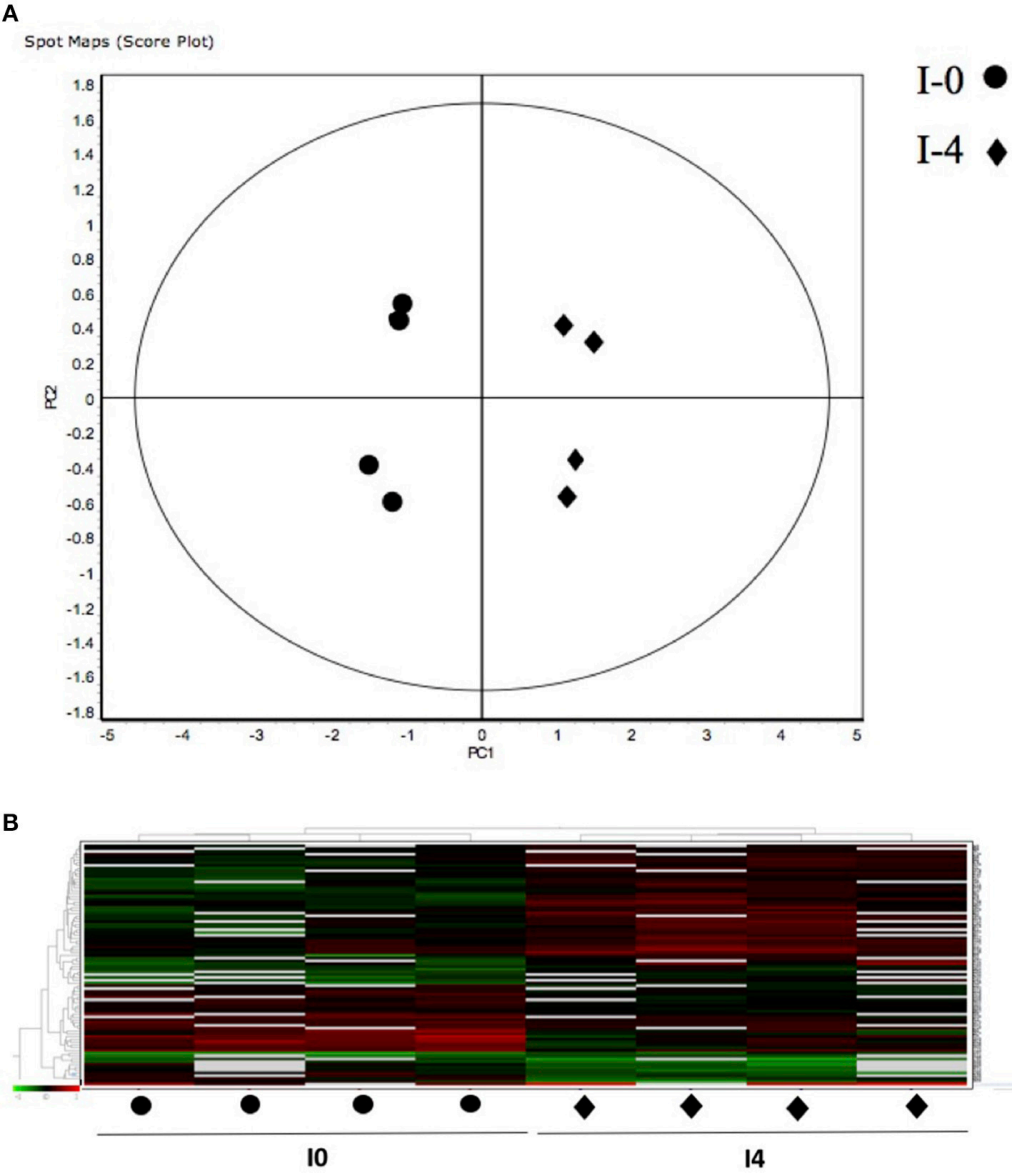
**(A)** Principal Component Analyses (PCA) and **(B)** hierarchical cluster analyses of the protein spots (sub-sets) differentially expressed of paralarvae of *Octopus vulgaris* corresponding to the condition 1 (I4 vs. I0) considered as function of dietary group and age (details in Supplementary Table 3). % of variance explained: PC1 = 66.6; PC2 = 11.4.

**Figure 3:**
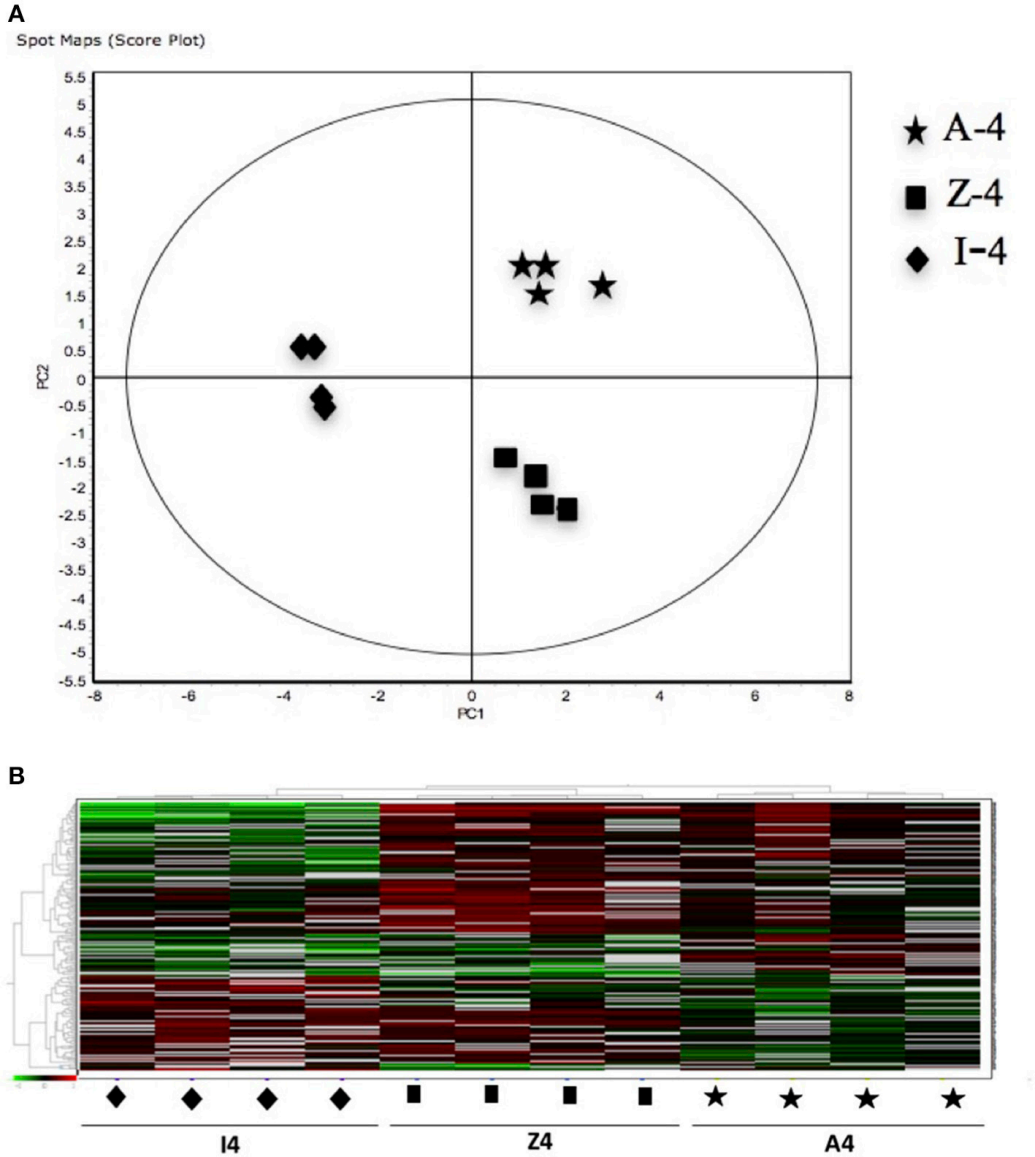
**(A)** Principal Component Analyses (PCA) and **(B)** hierarchical cluster analyses of the protein spots (sub-sets) differentially expressed of paralarvae of *Octopus vulgaris* corresponding to the condition 2 (A4, Z4 vs. I4) considered as function of dietary group and age (details in Supplementary Table 3). % of variance explained: PC1 = 73.7; PC2 = 9.6.

**Figure 4:**
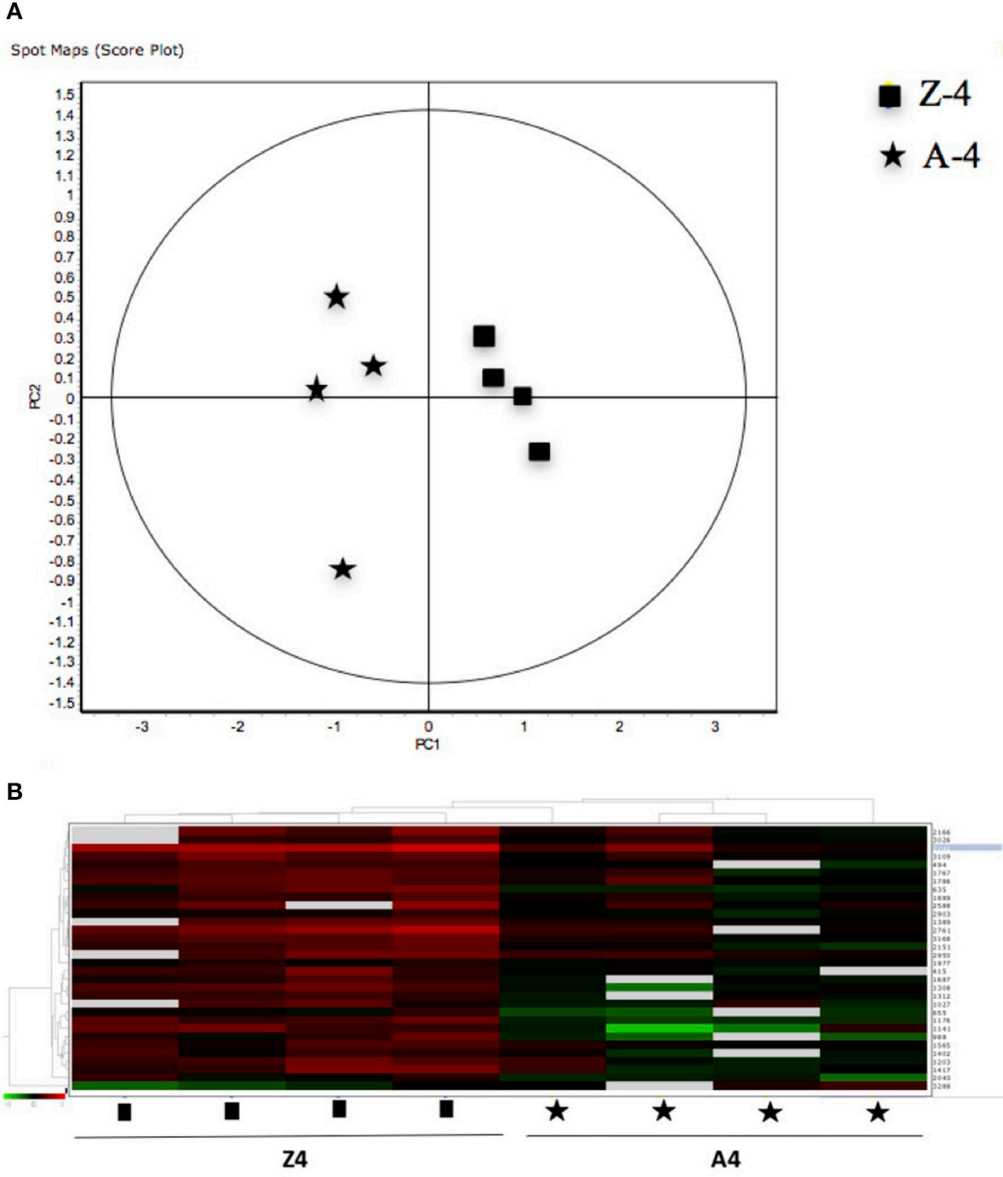
**(A)** Principal Component Analyses (PCA) and **(B)** hierarchical cluster analyses of the protein spots (sub-sets) differentially expressed of paralarvae of *Octopus vulgaris* corresponding to the condition 3 (Z4 vs. A4) considered as function of dietary group and age (details in Supplementary Table 3). % of variance explained: PC1 = 77.1; PC2 = 8.3.

**Figure 5:**
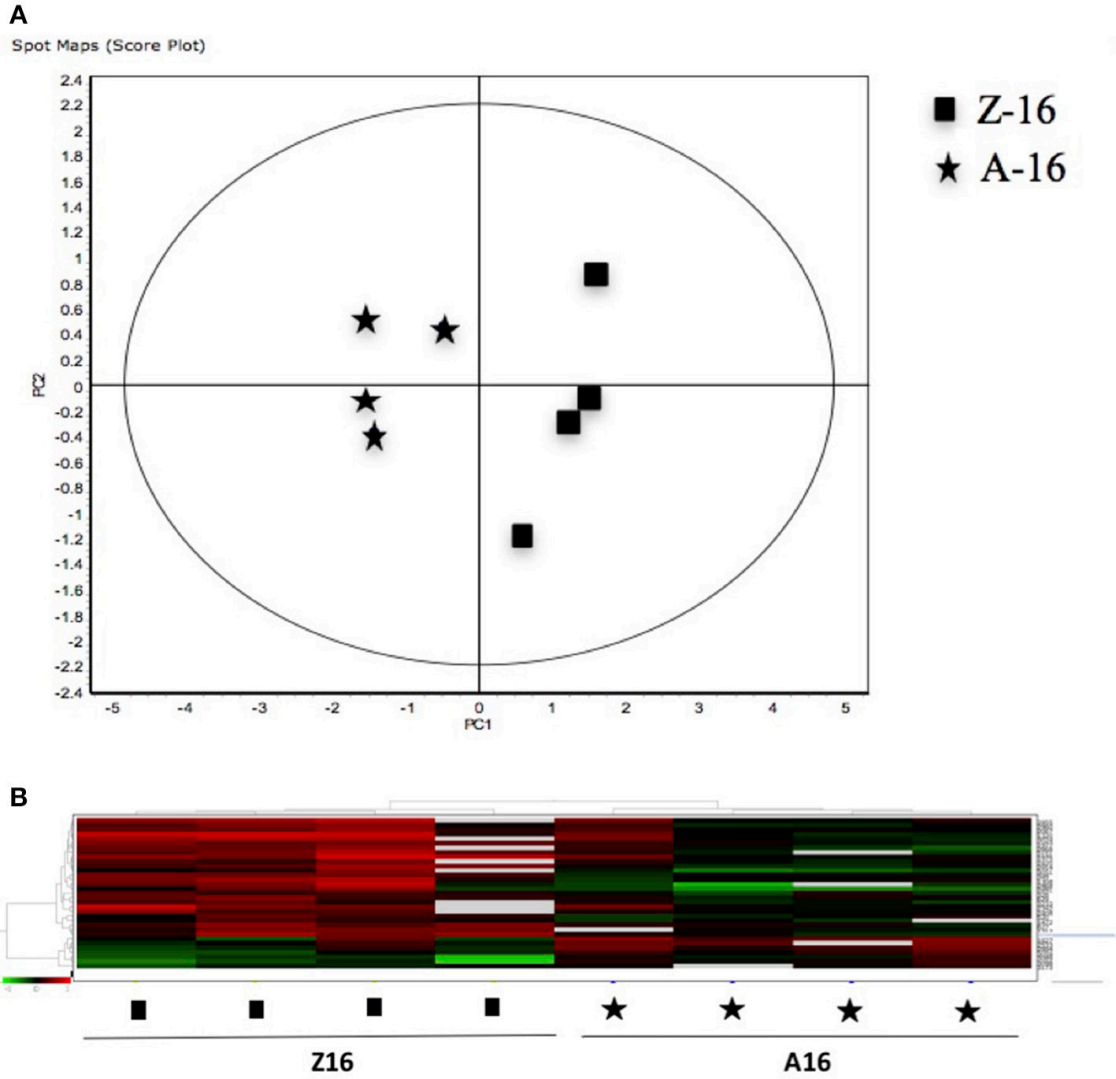
**(A)** Principal Component Analyses (PCA) and **(B)** hierarchical cluster analyses of the protein spots (sub-sets) differentially expressed of paralarvae of *Octopus vulgaris* corresponding to the condition 4 (Z16 vs. A16) considered as function of dietary group and age (details in Supplementary Table 3). % of variance explained: PC1 = 72.6; PC2 = 8.

The 12 gel images were subsequently analyzed using the DeCyder-DIA and BVA modules comparing the proteome of the 4 different conditions considered important as function of dietary group and age (Supplementary Table 3). Representative 2D-DIGE gels (gel master) of octopus paralarvae proteins from condition 1, comparing unfed 4 days old paralarvae (I4) with hatchlings (I0), is shown in Figure 1. A total of 4,507 proteins were detected in the gel master over the range of pH 3–11 NL and a molecular weight from approximately 10–250 kDa used in this study. BVA analyses revealed in condition 1 (I4 vs. I0), a total of 23 spots of proteins that showed significant changes in expression related with age (*t*-test, *p* ≤ 0.05). Among these protein spots, 11 were up regulated and 12 down regulated in the older unfed paralarvae (I4). From this set of proteins, it was possible to identify 16 proteins using mass spectrometry (Supplementary Tables 4–6). When fed and unfed 4 days old paralarvae (condition 2: A4, Z4 vs. I4) were compared, 24 proteins (22 up regulated and 2 down regulated) presented differential expression in fed paralarvae (one-way ANOVA, *p* ≤ 0.05). Of these, 15 protein spots were identified by mass spectrometry. In condition 3 (Z4 vs. A4) and 4 (Z16 vs. A16), where 4 and 16 days old paralarvae of both dietary groups were compared, a total of 10 (8 up regulated and 2 down regulated) and 7 protein spots (3 up regulated and 4 down regulated) respectively showed significant differences in expression (*t*-test, *p* ≤ 0.05) between zoeae and *Artemia* dietary groups. A total of 6 protein spots were identified in each condition by mass spectrometry. The 2D-DIGE analyses (spot no., *p*-value, fold change) and the results of protein identities differentially expressed in each condition are given in Table 1. Principal component analyses (PCA) and hierarchical cluster analyses of data for each condition are shown in Figures 2–5. The PCA results revealed a good separation of paralarvae groups from a specific sub-set of significant protein spots as function of diet and age in each condition. The dendogram after hierarchical cluster analyses also show a good separation of the spot maps as function of diet and age in each condition.

**Table 1:** **Protein identities differentially expressed in *Octopus vulgaris* paralarvae in the different conditions studied as function of dietary group and age**.

**Condition**	**Spot^a^ no**.	***P*-value^b^**	**Fold change^c^**	**Accession ID^d^**	**Protein name**	**Biological process**	**Species name for which protein was identified**
**1 (I4 vs. I0)**							
Up-regulated	2,535	0.0042	2.06	gi|268376225	–	–	–
	2,619	0.0190	2.01	gi|84447377	–	–	–
	2,668	0.0034	1.94	gi|2495111	S-crystallin 3	Structural constituent of eye lens	*Octopus vulgaris*
	2,711	0.0250	1.83	gi|84417280	–	–	–
	1,802	0.0290	1.71	gi|149688630	Glyceraldehyde-3-phosphate dehydrogenase (GAPDH)	Carbohydrate metabolism	*Octopus vulgaris*
	2,135	0.0074	1.60	gi|380324113	–	–	–
	1,229	0.0360	1.35	gi|9049272	Beta actin	ATP-binding	*Carassius auratus*
Down-regulated	912	0.0064	−1.20	gi|156407978	Predicted protein	–	*Nematostella vectensis*
	2,803	0.0170	−1.23	gi|84425674	–	–	–
	1,601	0.0003	−1.32	gi|342655056	–	–	–
	2,438	0.0110	−1.97	gi|378919614	–	–	–
	596	0.0091	−2.08	gi|158147451	Heat shock protein 70 kDa	Response to stress	*Segonzacia mesatlantica*
	1,645	0.0068	−2.20	gi|342662620	–	–	–
	2,600	0.0030	−2.23	gi|554913552	Triosephosphate isomerase	Carbohydrate metabolism	*Moniliophthora roreri* (MCA 2997)
	2,574	0.0280	−2.28	gi|565322954	Glutathione S-transferase 2	Detoxification	*Ophiophagus hannah*
	950	0.0350	−2.54	gi|342662199	–	–	–
**2 (A4, Z4** vs. **I4)**							
Up-regulated	2,742	0.00000021	19.50	gi|340742817	Arginine kinase	Energetic metabolism	*Amphioctopus fangsiao*
	2,592	0.0000056	11.96	gi|378905056	FO174848 whole embryos embryonic stages 16–28. cDNA clone ADY0AAA20YI14	–	*Sepia officinalis*
	2,745	0.00033	8.91	gi|268376271	gb|GT617746.1|GT617746	–	–
	2,571	0.0000099	8.81	gi|84443490	–	–	–
	3,059	0.00002	8.44	gi|2495111	S-crystallin 3	Structural constituent of eye lens	*Octopus vulgaris*
	3,098	0.0000041	7.72	gi|501292852	NADP^+^-specific isocitrate dehydrogenase	Energetic metabolism	*Riptortus pedestris*
	2,724	0.0005	4.29	gi|342650012	–	–	–
	2,558	0.00014	4.25	gi|518224143	Nitrilase	Nitrogen compound metabolism	*Bacillus endophyticus*
	3,050	0.000019	3.76	gi|508701	Actin	ATP-binding	*Cryptococcus neoformans* var. *grubii*
	1,827	0.00023	3.58	gi|391324898	Predicted: Actin, cytoplasmic 2-like isoform	ATP-binding	*Metaseiulus occidentalis*
	1,897	0.001	2.25	gi|350035483	Actin beta/gamma 1	ATP-binding	*Clonorchis sinensis*
	2,166	0.018	1.56	gi|149688630	Glyceraldehyde-3-phosphate dehydrogenase (GAPDH)	Carbohydrate metabolism	*Octopus vulgaris*
	1,889	0.043	1.30	gi|350035483	Actin beta/gamma 1	ATP-binding	*Clonorchis sinensis*
	2,151	0.019	1.04	gi|149688630	Glyceraldehyde-3-phosphate dehydrogenase (GAPDH)	Carbohydrate metabolism	*Octopus vulgaris*
Down-regulated	1,092	0.00033	−1.10	gi|556108726	hypothetical protein LOTGIDRAFT_231565	–	*Lottia gigantea*
**3 (Z4** vs. **A4)**							
Up-regulated	2,742	0.0056	2.66	gi|340742817	Arginine kinase	Energy metabolism	*Amphioctopus fangsiao*
	1,092	0.001	2.25	gi|556108726	hypothetical protein LOTGIDRAFT_231565	–	*Lottia gigantea*
	2,166	0.033	2.06	gi|149688630	Glyceraldehyde-3-phosphate dehydrogenase (GAPDH)	Carbohydrate metabolism	*Octopus vulgaris*
	2,151	0.0066	2.05	gi|149688630	Glyceraldehyde-3-phosphate dehydrogenase (GAPDH)	Carbohydrate metabolism	*Octopus vulgaris*
	1,889	0.023	1.52	gi|350035483	Actin beta/gamma 1	ATP-binding	*Clonorchis sinensis*
Down-regulated	2,558	0.01	−1.73	gi|518224143	Nitrilase	Nitrogen compound metabolism	*Bacillus endophyticus*
**4 (Z16 vs. A16)**							
Up-regulated	2,151	0.013	3.21	gi|149688630	Glyceraldehyde-3-phosphate dehydrogenase (GAPDH)	Carbohydrate metabolism	*Octopus vulgaris*
Down-regulated	2,724	0.041	−1.15	gi|342650012	–	–	–
	3,059	0.011	−2.27	gi|2495111	S-crystallin 3	Structural constituent of eye lens	*Octopus vulgaris*
	1,827	0.037	−2.41	gi|391324898	Predicted: Actin, cytoplasmic 2-like isoform	ATP-binding	*Metaseiulus occidentalis*
	2,558	0.021	−2.65	gi|518224143	Nitrilase	Nitrogen compound metabolism	*Bacillus endophyticus*
	3,098	0.017	−2.85	gi|501292852	NADP-specific isocitrate dehydrogenase	Energetic metabolism	*Riptortus pedestris*

a *Spot numbering*.

b *Student t-test P-value*.

c *Average fold change ratio in each condition as calculated by DeCyder BVA analysis*.

d *Protein accession number from NCBI*.

*− Denotes non-identified*.

A total of 55 spots that showed significant differences in the experimental groups after gel image analyses, and with enough quantity of protein to be manually excided from analytical gels, were selected for protein identification by MS. From these, 43 protein spots were successfully identified in public databases. Different protein spots were identified as the same protein due post-translational modifications. Some of the identified proteins were found to be involved in several metabolic pathways, related with carbohydrate metabolism [glyceraldehyde-3-phosphate-dedydrogenase (GAPDH), triosephosphate isomerase], and other pathways of energetic metabolism (NADP+-specific isocitrate dehydrogenase and arginine kinase). Other identified proteins were related with detoxification [glutathione-S-transferase (GST)], stress [heat shock proteins (HSP70)], structural constituent of eye lens (S-crystallin 3), and cytoskeleton (actin, actin-beta/gamma1, and beta actin). Table 2 summarizes the main changes in expression of the above-mentioned proteins in the different conditions studied as function of dietary group and age. It is interesting to note the up-regulation of GAPDH, a key protein in carbohydrate metabolism, in all conditions, with the 16 days old zoeae fed group (Z16, condition 4) showed the highest fold change (3.21-fold). In condition 1, unfed paralarvae (I4) presented 2 proteins involved in carbohydrate metabolism, one of which was up regulated (GAPDH), whereas another (triosephosphate isomerase) was down regulated. Other 2 up regulated proteins were associated with vision function (S-crystallin 3, by over ~2-fold) and cytoskeletal structure (beta actin, increased 1.35-fold). Finally, another 2 down regulated (by over ~2-fold) proteins were related with stress (HSP70) and detoxification (GST). Four days old fed paralarvae (A4 and Z4, condition 2) showed an increase in abundance in all the proteins identified, except for the spot 1,090 (hypothetical protein LOTGIDRAFT_231565), which decreased by ~1-fold. Also, it is to note that in fed paralarvae groups there was overexpression of proteins involved in energy metabolism (arginine kinase, 19.5-fold) and NADP+-specific isocitrate dehydrogenase, 7.72-fold) and vision (S-crystallin 3, 8.44-fold) compared with unfed paralarvae group (I4). Moreover, an increase in a protein involved in nitrogen metabolism (nitrilase, 4.25-fold), and in two isoforms of actin (actin, 3.76-fold and actin beta/gamma I, 2.25-fold) was identified in fed paralarvae groups. When both dietary groups were compared (condition 3 and 4), the common proteins identified with differential expression, showed also similar changes in abundance. Interestingly, most of the proteins showing significant changes were up regulated in 4 days old paralarvae, while the opposite occurs in 16 days old paralarvae. It is to note that in Z4 group (condition 3), two of the three proteins involved in energy metabolism (GAPHD and arginine kinase) increased, whereas the third (NADP+-specific isocitrate dehydrogenase) decreased. S-crystallin3 decreased only (2.27-fold) in Z16 group (condition 4).

**Table 2:** **Summary of the fold changes in expression of protein identities differentially expressed in *O. vulgaris* paralarvae in the different condition studied as function of dietary group and age**.

**Protein name**	**Condition 1 I4 vs. I0**	**Condition 2 A4, Z4 vs. I4**	**Condition 3 Z4 vs. A4**	**Condition 4 Z16 vs. A16**
S-crystallin 3	↑ (1.94)	↑ (8.44)		↓ (−2.27)
Glyceraldehyde3-phosphate dehydrogenase (GAPDH)	↑ (1.71)	↑ (1.56) ↑ (1.04)	↑ (2.06) ↑ (2.05)	↑ (3.21)
Beta actin	↑ (1.35)			
Heat shock protein 70KDa	↓ (−2.08)			
Triosephosphate isomerase	↓ (−2.23)			
Glutahione-S-transferase 2	↓ (−2.28)			
Nitrilase		↑ (4.25)	↓ (−1.73)	↓ (−2.56)
NADP^+^specific isocitrate dehydrogenase		↑ (7.72)		↓ (−2.85)
Actin		↑ (3.76)		
Actin beta/gamma1		↑ (2.25)	↑ (1.52)	
Arginine kinase		↑ (19.5)	↑ (2.66)	

*↑, up-regulated; ↓, down-regulated in each condition studied. ( ), fold change values. I, unfed group; A, Artemia group; Z, zoeae group. Number indicates age (days). vs., versus*.

## Discussion

Common practice in *O. vulgaris* paralarval rearing is to use spawns from single females. This is practically unavoidable because *O. vulgaris* is a semelparous species, and under the current state of the art, broodstock has to be captured from the wild, each with a different feeding and maturation background. Once captured, each female spawns at different times, and the egg masses undergo a long embryonic phase, making almost impossible to synchronize different hatchings. One point to consider then, is whether the molecular behavior of the offspring is linked to the genetic background of the female in particular. It has to be noted, however, that intercalibration approaches carried out among different laboratories (Garrido et al., 2017) and meta-analysis studies (Garrido et al., 2016a) reveal that above of the unavoidable variability inherent to the geographical origin and rearing scenarios, most rearing problems and physiological responses of the paralarvae are repetitive.

As far as we know, this is the first study using a proteomic approach to analyze the effect of diet and to search novel biomarkers of nutritional stress on octopus paralarvae. The proteome of octopus paralarvae according to the multivariate analyses (PCA and hierarchical), showed that changes in specific sub-sets of proteins differentially expressed as function of diet and age, allowed to describe characteristic proteomes for each experimental condition (see Figures 2–5). Similar result has been previously reported by Sveinsdóttir and Gudmundsdóttir (2010) for Atlantic cod (*Gadus morhua*) larvae using proteome analysis to study feeding effects. These authors found changes in abundance in a sub-set of 13 protein spots in the proteome of cod larvae fed with saithe (*Pollachius virens*) protein hydrolysate (SPH). Among the proteins identified here, it is noteworthy that most are involved in energy metabolism such as glyceraldehyde-3-phosphate dehydrogenase (GAPDH), triosephosphate isomerase, NADP+-specific isocitrate dehydrogenase, and arginine kinase. This is not surprising since, early development of many marine organisms undergoing larval phases, and particularly of octopus paralarvae is highly dependent on energy for growth (von Boletzky and Villanueva, 2014). Other proteins identified are involved in several cellular processes related with detoxification (GST), stress [heat shock proteins (HSP70)], vision (S-crystallin 3) and cytoskeleton (actin, actin-beta/gamma1 and beta actin), physiologically crucial during early life stages of development.

The enzyme GAPDH has been known mainly as a “housekeeping” protein (Barber et al., 2005), with a role as key intermediate component of glycolysis and as a source of NADH. In fact, GADPH transforms glyceraldehyde-3-phosphate to glycerate 1,3-bisphospate and mediates the formation of NADH and ATP. The up-regulation of GAPDH in all conditions analyzed in our work could be related with these functions. Paralarval stages show a very active metabolism and fast growth, so obtaining energy and reducing power are essential. Besides, GADPH has demonstrated to be a multifunctional protein at least in vertebrates (Sirover, 1999; Baumgarner et al., 2012), displaying diverse activities including membrane, cytoplasmic and nuclear functions in endocytosis, mRNA regulation, DNA replication and repair, as well as regulation of apoptosis, as has been demonstrated in mammals (Sirover, 1997, 2011; Tristan et al., 2011). GAPDH expression has also been associated with exposure to hypoxia or anoxia and temperature variation in fish (Smith et al., 2009; Baumgarner et al., 2013; Jayasundara et al., 2015), and in mammals with a function of the cell proliferative state (Meyer-Siegler et al., 1992; Mansur et al., 1993; Gong et al., 1996). This last fact could also be the common origin of the over expression of GAPDH found in paralarvae of all conditions, considering the rapid growth undergone in this phase of the biological cycle, with amino acids as the main source of energy (Lopes et al., 2016) and glycolysis playing a secondary role in paralarvae development (Lee, 1994; Navarro et al., 2014). It is important to note, however, that in the early days posthatching paralarvae use yolk reserves. These include glycogen and triglycerides (Quintana et al., 2015). In both cases substrate is supplied for the GAPDH, either by glycolysis or by the incorporation of the glycerol from mono or triglycerides into the glycolytic pathway, as evidenced by the glycerol kinase (GyK) activity found in newly hatched paralarvae (Cardenete et al., 2016).

In condition 1, unfed 4 days old paralarvae showed up-regulation of S-crystallin 3 respect to hatchlings. This protein that is a structural constituent of the lenses of the eyes in octopus. S-crystallins are soluble proteins in eye lens that contribute to the transparency and optical clarity (De Jong et al., 1989). The over-expression of this protein involved in vision, shows the importance of eyesight in paralarvae from the very beginning of their development, even under starving conditions. Although paralarvae are provided with chemoreceptors, they are mainly visual hunters (Lee, 1994; von Boletzky and Villanueva, 2014). Hatchlings enter in a planktonic phase, where they need to start feeding from the first day after hatching, and the active search (hunt) for food has been recorded 2 days after hatch at temperatures between 18 and 20°C (Iglesias et al., 2006), even before the yolk reserves are exhausted (Villanueva and Norman, 2008).

Regarding energetic metabolism, in condition 1 it can be observed how triosephosphate isomerase (TPI) is down regulated in 4 days starved paralarvae with respect to hatchlings. This enzyme catalyzes the reversible transformation of dihydroxyacetone phosphate (DHAP) to glyceraldehyde 3-phosphate (GAP) in the glycolysis pathway. DHAP is mainly provided from glucose or glycerol from triglycerides. Thus, this fact points to a decrease in energy production by glycolysis and/or catabolism of triglycerides, which is in agreement with the fasting situation of paralarvae, and with the foreseeable exhaustion of the yolk reserves which, under normal conditions, are completely depleted around 4–5 days after hatching (Nande et al., 2017)

Usually the enzymatic reaction catalyzed by TPI runs to the formation of GAP, due that this one is rapidly eliminated by the following reaction of the glycolytic pathway that is catalyzed by the GAPDH enzyme (Blacklow et al., 1988; Harris et al., 1998). In this case, GADPH is lightly over regulated and therefore, there is a contrasting situation that should lead to a decrease of the GAP substrate. Obviously, in this case, the over expression of GADHP found, would be more related with the other functions of this enzyme mentioned above (i.e., NADH production) than with the obtention of energy by glycolysis.

Beta actin is also up-regulated in unfed 4 days old paralarvae which is part of the components of the cell cytoskeleton. This protein is highly conserved and is involved in support and different types of cell motility. Cephalopods, especially at paralarvae stages, display high growth rates, and proteins of cytoskeleton may be of paramount importance during their development.

HSP70 was down-regulated in unfed octopus paralarvae. These results are in line with a previous study, where starvation was related with decreased HSP70 levels in 4 days old octopus paralarvae, whereas increased HSP70 levels were detected in fed paralarvae (Varó et al., 2013). Previous studies on early life stages, mainly in fish, indicate variability in HSP70 response related to starvation. In fact, increased (Cara et al., 2005), decreased (Deng et al., 2009), or unchanged (Han et al., 2012) HSP70 levels have been found in fish larvae related with starvation or during food restriction. HSP70 is a chaperone protein that assists in the folding and transport of other proteins, and thus can be critical in periods of rapid growth (Kiang and Tsokos, 1998). Thus, its expression can be altered by several stressors, including nutritional stress, caused by starvation. High growth rates in cephalopods are based in increasing body mass by protein synthesis and accretion, especially at paralarvae stages, that require large amounts of proteins and amino acid in the diet to satisfy the energy demands (Navarro et al., 2014). In larval fish, starvation is associated to amino acid restriction and enhanced proteolysis that affect cellular protein homeostasis, which translates into lower growth and survival (Conceição et al., 1997). For example, in white sturgeon larvae (*Acipenser transmontanus*) starvation reduced the induction of HSPs (HSP70 and HSP90), and decreased the body weight (Han et al., 2012). The growth (dry weigh) of unfed paralarvae group was much lower (0.177 ± 0.070 mg), compared to hatchlings (0.333 ± 0.080 mg) and 4 days old fed paralarvae groups (Z: 0.411 ± 0.014 mg; A: 0.367 ± 0.033 mg), with a loss of their initial weigh around 50% (47.7%). In agreement with former larval fish observations, the down-regulation of HSP70 found in starved paralarvae could be related with lower metabolic rates, linked with restriction of protein and amino acid anabolism, resulting in a lower or poor growth.

GST also showed down-regulation in unfed paralarvae. This enzyme is implicated in the detoxification of reactive electrophilic compounds and xenobiotic substances as antioxidant defense. Although changes of antioxidant defense have been associated with aging in invertebrates, including cephalopods (Zielinski and Pörtner, 2000; Barata et al., 2005), the accumulation of defective macromolecules caused by age has been related with increase of oxidative damage and/or by loss of the ability to repair or degradate these molecules (Stadtman, 1992, in Zielinski and Pörtner, 2000). Our results suggest that the decrease in GST could lead to the accumulation of electrophilic molecules indicating a detrimental effect on starved paralarvae. This, together with the decrease in HSP70, also involved in cellular defense, would constitute a response to an advanced stress scenario in unfed paralarvae. However, the lower metabolic rate probably produced by fasting could also reduce the levels of GST, as pointed out by Morales et al. (2011). In fish the transcriptional response of GST to starvation varies according to tissue, species and fasting length. Thus, while in cod muscle (*G. morhua*) GST does not show modifications, in liver GST increases after short periods of fast in rock bream (*Oplegnatus faciatus*), whereas in rainbow trout (*Onconhynchus mykiss*) GST decreases in fish starved for 3 or more weeks (Morales et al., 2011). Interestingly, GST is related with S-crystallin in cephalopods. In fact, as pointed out by Tomarev et al. (1991) the use of detoxification stress proteins like GST and aldehyde dehydrogenase as cephalopod crystallins, is indicative of a common strategy for recruitment of enzyme-crystallins during the convergent evolution of vertebrate and invertebrate lenses. A recent study on *O. vulgaris* has revealed that the loss of GST enzyme activity in lens tissue is linked to the enhanced protein stability of S-crystallin via glutathione binding (Tan et al., 2016). In the light of these data, and although protein identification in our study was not carried out at the cellular location level, it is tempting to suggest that the decrease of GST might also be indicative of possible alterations in the lens structure of starved paralarvae.

In 4 days old fed paralarvae (condition 2), all proteins showing differential expression respect to unfed group were over expressed. It is interesting to highlight particularly the high over- expression of S-Crystalin 3 (8.44-fold change) that is in line with the former responses of this protein in 4 days old starving paralarvae, and support the crucial importance of eyes (vision). Particularly, S-Crystalin proteins, for their role in the refractive properties of the eye lens (Tomarev et al., 1991; Tomarev and Piatigorsky, 1996) may be of paramount importance in active visual hunters like octopuses for their adequate feeding and growth, especially during the first days of development, when adequate provision of essential nutrients is necessary for their rapid buildup. Thus, eye structure and function may be modulated by the feeding/nutritional (fed vs. starving) status of paralarvae and “vice versa.”

Actin and actin beta/gamma1, cytoskeleton proteins, showed high increases in their expression in 4 days old fed groups respect to unfed ones, paralleling the high growth (dry weight, see above) and pointing at their importance in the development.

Nitrilase is also up-regulated in fed 4 days old paralarvae. This protein is part of a superfamily consisting in thiol enzymes involved in biosynthesis and post-translational modification of natural product in plants, animals, and fungi (Pace and Brenner, 2016). In this study, the sequence producing significant alignment of this protein (NCBI gi| 518224143) corresponds to a nitrilase described in *Bacillus endophyticus*. Bacterial nitrilases are used for biochemical synthesis and for environmental remediation (Cowan et al., 1998). A recent study has shown that *B. endophyticus* compared to different *Bacillus* strains has more genes related with energetic and transport metabolism of carbohydrates and lipids, than those related to cell envelope biogenesis and signal transduction mechanisms (Jia et al., 2015). This could suggest that the up-regulation of nitrilase could be linked to the bacterial metabolism that accompanies the gut microbiome of fed paralarvae, more than with the paralarval metabolism itself.

The high up regulation (7.72-fold change) of an isocitrate dehydrogenase isozyme dependent of NADP^+^ (NADP-IDH), found in 4 days fed paralarvae (condition 2), point to a high activity of the Krebs cycle (TCA). In eukaryotes, there are three isozymes of IDH, two located in the mitochondrial matrix (NAD and NADP dependent respectively) related with energy production by TCA cycle and another one NADP dependent IDH in cytosol, which provides NADPH. The latter is necessary for the maintenance of the reduced glutathione and peroxiredoxin antioxidant systems by mean of the enzymes glutathione reductase (GR) and thioredoxin reductase (TrxR) respectively (Xu et al., 2004; Tomanek and Zuzow, 2010; Tomanek, 2015). Both systems are required to prevent the deleterious effect of reactive oxygen species (ROS). In condition 2, fed paralarvae seem to have a high aerobic metabolism and an up regulation in the production of NADPH. To account for this production, it is necessary to provide continuously substrate to the IDH catalyzed reaction. In short, sufficient citrate synthesis should be produced by a previous reaction of the Krebs cycle catalyzed by the enzyme citrate synthase. Therefore, a sustained supply of citrate synthase substrates: oxaloacetate and acetyl coenzyme A, are required (Tomanek and Zuzow, 2010). The results of Cardenete et al. (2016) seem to support this. In fact, 3 days old octopus paralarvae (fed *Artemia*) showed a significant increase in glutamate oxaloacetate transaminase activity (GOT, enzyme that produce oxaloacetate) as well as an increase in the activity of β-hydroxyacyl CoA dehydrogenase (HOAD), a key enzyme in beta oxidation of fatty acids, process that produces acetyl CoA. Both activities are also indicative of a high level of aerobic metabolism.

With regard to arginine kinase, the enzyme that shows the higher up regulation (19.5-fold), it catalyzes the first step of the arginine phosphate system that provides anaerobic fuel in several invertebrates including cephalopods (Fields et al., 1976; Gäde, 1980; Ellington, 2001). The system produces ATP in a first step breaking arginine phosphate, stored in mollusk muscles (Regnouf and van Thoai, 1970), by means of the activity of arginine kinase (AK), producing arginine and ATP. In a second step the condensation of the amino acid arginine with pyruvate takes place. This reaction produces octopine and it is catalyzed by the NADH dependent enzyme octopine dehydrogenase (ODH; Fields et al., 1976). In this context, the over expression of GAPDH found may be explained, since it provides the NADH required by AK activity.

The function of this anaerobic way of obtaining energy is related in paralarvae with episodes of physiological hypoxia in muscle when there is a great demand of energy as it happens in fast and intensive swimming (Baldwin, 1982; Lee, 1994). Indeed, it is associated to prey capture by paralarvae, that involves bursts of swimming energetically very expensive (Baldwin, 1982). In agreement with these data, octopine dehydrogenase activity shows a significant increase in the first days of paralarvae life (Cardenete et al., 2016), indicating an increase in the predatory capacity of the paralarvae with their development.

In conditions 3 and 4, when both dietary groups are compared (see Table 2), proteins with differential expression showed similar changes in their abundance, suggesting that they were most probably related to development (age) than to diet. The two diets supplied were more different in qualitative than in quantitative composition, with the co-feeding treatment providing essential lipids in comparison with the diet based solely in *Artemia*. In fact, every effort was made to adjust food so that there was equivalent prey density in both dietary treatments, hence the co-feeding schedule. All these suggestions, however must be contemplated with care since working with live preys is always difficult to handle and control. Suffice to say that octopus paralarvae are active selective predators as has been reported recently (Roura et al., 2012). It must be pointed out however, that until the nutritional requirements of the paralarvae are well established, helping to design and produce adequate inert diets, live food remains the best alternative. Experimental designs focused on unveiling differential expressions of proteins linked to specific aspects of food composition should be foreseen in future works to help to cope with these aspects, but were beyond the scope of the present work.

It is to note that in the zoeae fed group, S-crystallin 3 showed significant change only in 16 days old paralarvae (Z16, condition 4), decreasing by 2.27-fold. In view of all the changes of this protein in the different condition studied (see Table 2), one would conclude that starvation seems to affect more to the eye lens structure in 16 days old paralarvae fed with zoeae than with *Artemia*. As mentioned above, alterations in the eye lens structure, lower transparency and optical clarity should produce altered vision and less success in hunting and feeding. This should translate in lower growth. Although Z16 paralarvae showed higher DW (0.713 ± 0.062 mg) and specific growth rate (SGR, % DW day^−1^: 4.961 ± 0.58) compared with *Artemia* fed group (DW: 0.621 ± 0.051 mg; SGR: 4.043 ± 0.55), as previously discussed by Garrido et al. (2016a), no significant differences in growth were found between both dietary groups. Although it is always delicate to make direct comparisons due to differences in methodologies and feeding schemes (aggravated by the use of live preys), as well as variability in spawn quality (Garrido et al., 2017), it is to note that other trials using zoeae as food have reported higher growth (Iglesias et al., 2004, 2014; Carrasco et al., 2006; Iglesias and Fuentes, 2014). Thus, bearing in mind that adequate prey density at this stage of development is critical (Villanueva et al., 2002), the decrease in S-crystallin 3 in zoeae group could be a response of paralarvae to starvation, more than to the qualitative composition of the diet. This protein would act then as a biomarker of starvation pointing at this condition in the co-feeding treatment as it did in 4 days old starved paralarvae. In fact, co-feeding was used because there was not guarantee of enough provision of zoeae to fulfill the necessary prey density, and paralarvae are selective feeders.

The down-regulation of nitrilases found in the zoeae group at 4 and 16 days (condition 3 and 4) compared to the *Artemia* group suggest, in agreement with the former observations (condition 2) linking these proteins to bacterial nitrilases metabolism present in the gut microbiome of fed paralarvae, that this bacterial metabolism was lower in co-fed paralarvae, perhaps in line with a scarcity of food scenario.

The up-regulation of cytoskeleton proteins, actin and actin beta/gamma1, together with the increase in arginine kinase, involved in energy production (ATP) found only in 4 days old paralarvae fed with zoeae, supports the importance of structural and energetic proteins in the enhanced growth of this group compared with *Artemia* in periods of high energy demand as occurs in the first 4 days of the octopus' development. On the other hand, the down regulation of NADP^+^ specific isocitrate dehydrogenase suggests a shift toward anaerobic metabolism in 16 days old paralarvae fed with zoeae, as compared with the high up regulation found in 4 days old fed paralarvae.

Overall, the results show that in 4 days old zoeae fed paralarvae, all the proteins identified were up regulated, which could be associated with higher growth (DW) than in the case of those *Artemia* fed. On the contrary, at 16 days the proteins identified were down regulated in the zoeae group, except GAPDH that was always up regulated in all the conditions studied. The consistency of the up-regulation in the rest of the different feeding conditions suggests that the decrease in the zoeae group at 16 days might represent an indication of nutritional stress in paralarvae linked to insufficient availability of food.

## Conclusion

In summary, the results obtained in the pattern of protein expression allow defining characteristic proteomes of paralarvae of *O. vulgaris* depending on diet and age. This study showed that proteome of *O. vulgaris* paralarvae is affected by fasting during the first 4 day of development, with proteins like S-crystallin, arginine kinase and NAD^+^ specific isocitrate dehydrogenase showing the highest over expression in fed respect to unfed paralarvae. When both *Artemia* and *Artemia* plus zoeae dietary groups are compared, the up regulation of the proteins identified in 4 days old zoeae fed paralarvae, followed by the decrease found at 16 days old, might suggest an indication of nutritional stress in this group linked to starvation in a sub-optimal prey concentration scenario, more than with the quality of the diet. Although co-feeding is currently a good strategy to balance the quality of diet for achieving the best growth and survival in on growing octopus paralarvae during the first days of development, later around16 days of development, every effort has to be made to supply adequate amounts of food. Overall, the changes in the abundance of proteins like S-crystallin, arginine kinase and NAD^+^ specific isocitrate dehydrogenase may be used as novel biomarkers to assess the nutritional status of octopus paralarvae. Their increase/decrease in abundance may be related to fed vs. starving paralarvae, particularly in the first 4 days of development. Proteomics techniques represent not only an invaluable approach to go beyond the “state of evidence” in the search for the causes of poor performance of paralarval cultures at present, but are also a promising tool for fine tuning welfare once the main problems and constraints are overcome.

## Author contributions

IV: Design of proteomic procedures, analysis, and interpretation of the findings, and draft and writing of the manuscript. GC: Interpretation of the findings, writing discussion (metabolism) and revision of the manuscript. OM and FH: Analysis and interpretation of the findings, and general writing of the manuscript. JI and JO: Octopus paralarvae cultures and execution of the experiments. EA: Design experiments and revision of the manuscript. JN: Interpretation of the findings, writing and revision of the manuscript.

## Funding

This study was funded by the “Ministerio de Economía y Competividad (Spanish Government)” under the projects OCTOPHYS (AGL-2010-22120-CO3-02) and OCTOWELF (AGL2013-49101-C2-2-R), and by the “Generalitat Valenciana” under the project PROMETEO II/2014/085.

### Conflict of interest statement

The authors declare that the research was conducted in the absence of any commercial or financial relationships that could be construed as a potential conflict of interest.
