# Supplementary material for: Dietary Effect on the Proteome of the Common Octopus (Octopus vulgaris) Paralarvae
Source: Front Physiol. 2017 May 17;8:309. doi: 10.3389/fphys.2017.00309 (PMC5434110; doi:10.3389/fphys.2017.00309)
Supplement: Supplementary file 1 [file Table1.docx]

**Supplementary Table 1.** Experimental dietary groups used for differential in-gel electrophoresis (DIGE) analyses.

| **Group** | **Age (days)** | **Dietary group** | **Sample** |
| --- | --- | --- | --- |
| Group 1 | 0 | I0 | I0.1, I0.2, I0.3, I0.4 |
| Group 2 | 4 | I4 | I4.1, I4.2, I4.3, I4.4 |
| Group 3 | 4 | A4 | A4.1, A4.2, A4.3, A4.4 |
| Group 4 | 4 | Z4 | Z4.1, Z4.2, Z4.3, Z4.4 |
| Group 5 | 16 | A16 | A16.1, A16.2, A16.3, A16.4 |
| Group 6 | 16 | Z16 | Z16.1, Z16.2, Z16.3, Z16.4 |

(I): unfed group; (A): Artemia group; (Z): zoeae group.

The number after the dietary group in the sample column indicates the number of the sample.
