# Supplementary material for: Dietary Effect on the Proteome of the Common Octopus (Octopus vulgaris) Paralarvae
Source: Front Physiol. 2017 May 17;8:309. doi: 10.3389/fphys.2017.00309 (PMC5434110; doi:10.3389/fphys.2017.00309)
Supplement: Supplementary file 2 [file Table2.docx]

**Supplementary Table 2.** Experimental design for differential in-gel (DIGE) of octopus paralarvae proteins.

| **Gel** | **Cy2** | **Cy3** | **Cy5** |
| --- | --- | --- | --- |
| Gel 1 | Internal standard | I0.1 | I4.1 |
| Gel 2 | Internal standard | I0.2 | I4.2 |
| Gel 3 | Internal standard | I4.3 | I0.3 |
| Gel 4 | Internal standard | I4.4 | I0.4 |
| Gel 5 | Internal standard | A4.1 | A16.1 |
| Gel 6 | Internal standard | A4.2 | A16.2 |
| Gel 7 | Internal standard | A16.3 | A4.3 |
| Gel 8 | Internal standard | A16.4 | A4.4 |
| Gel 9 | Internal standard | Z16.1 | Z4.1 |
| Gel 10 | Internal standard | Z16.2 | Z4.2 |
| Gel 11 | Internal standard | Z4.3 | Z16.3 |
| Gel 12 | Internal standard | Z4.4 | Z16.4 |

(I): unfed group; (A): Artemia group; (Z): zoeae group.

The number after the dietary group in Cy3 and Cy5 columns indicates the number of the sample
