# Supplementary material for: Dietary Effect on the Proteome of the Common Octopus (Octopus vulgaris) Paralarvae
Source: Front Physiol. 2017 May 17;8:309. doi: 10.3389/fphys.2017.00309 (PMC5434110; doi:10.3389/fphys.2017.00309)
Supplement: Supplementary file 3 [file Table3.DOCX]

**Supplementary Table 3.** Conditions analysed using DeCyder-DIA software comparing the octopus proteomes considered important as function of dietary group and age.

| **Condition** | **Comparison** | **Dietary group** | **Age (days)** |
| --- | --- | --- | --- |
| 1 | I4 ***vs*** I0 | I | 0, 4 |
| 2 | A4, Z4 ***vs*** I4 | I, A, Z | 4 |
| 3 | Z4 ***vs*** A4 | A, Z | 4 |
| 4 | Z16 ***vs*** A16 | A, Z | 16 |

(I): unfed group; (A): *Artemia* group; (Z): zoeae group.

***vs*** : versus
